# Activity of β-Lactamase Inhibitor Combinations Against Enterobacterales Isolated from Patients with Intra-Abdominal Infection from United States Medical Centres (2019–2023)

**DOI:** 10.3390/antibiotics14060544

**Published:** 2025-05-27

**Authors:** Helio S. Sader, John H. Kimbrough, Marisa L. Winkler, Rodrigo E. Mendes, Mariana Castanheira

**Affiliations:** JMI Laboratories, Element Iowa City, North Liberty, IA 52317, USA; hank.kimbrough@element.com (J.H.K.); marisa.winkler@element.com (M.L.W.); rodrigo.mendes@element.com (R.E.M.); mariana.castanheira@element.com (M.C.)

**Keywords:** aztreonam-avibactam, beta-lactamase inhibitor combination, antimicrobial resistance, carbapenem-resistant Enterobacterales, CRE, MDR

## Abstract

**Objective**: To evaluate the antimicrobial susceptibility of Enterobacterales isolated from patients with intra-abdominal infections (IAI) in United States (US) medical centres. **Methods**: A total of 2036 isolates (1/patient) were consecutively collected from patients with IAI in 63 US hospitals in 2019–2023 and susceptibility tested by broth microdilution. Carbapenem-resistant Enterobacterales (CRE) were screened for carbapenemases by whole genome sequencing. **Results**: The most common Enterobacterales species were *E. coli* (47.1%), *K. pneumoniae* (18.7%), and *E. cloacae* species complex (9.8%). The most active agents were aztreonam-avibactam (MIC_50/90_, ≤0.03/0.12 mg/L), ceftazidime-avibactam (MIC_50/90_, 0.12/0.25 mg/L), and meropenem-vaborbactam (MIC_50/90_, 0.03/0.06 mg/L) with 99.9% susceptibility. A multidrug-resistant (MDR) phenotype (nonsusceptibility to ≥3 classes) was observed in 21.4% of Enterobacterales (*n* = 436). Piperacillin-tazobactam was active against 87.2% of Enterobacterales overall and 50.2% of MDR isolates, and meropenem was active against 99.2% of Enterobacterales and 96.1% of MDR isolates. Only 51.6% of Enterobacterales were susceptible to ampicillin-sulbactam. An acquired broad-spectrum β-lactamase gene was identified in 207 (10.2%) isolates and included extended-spectrum β-lactamases (ESBL; *n* = 182), transferable AmpC (*n* = 24) and carbapenemases (*n* = 9). Eight isolates produced two β-lactamase classes. **Conclusions**: Aztreonam-avibactam, ceftazidime-avibactam, and meropenem-vaborbactam exhibited almost complete activity (99.9% susceptibility) against Enterobacterales causing IAI in US hospitals. In contrast, piperacillin-tazobactam exhibited limited activity against these organisms, especially those with a MDR phenotype.

## 1. Introduction

Intra-abdominal infections (IAIs) represent a common cause of admission in United States (US) hospitals and is associated with elevated morbidity and mortality. IAI can take several forms. Infection may be in the retroperitoneal space or within the peritoneal cavity. Intraperitoneal infection may be diffuse or localized into one or multiple abscesses. Intra-abdominal abscesses may form in dependent recesses such as the pelvic space or Morrison’s pouch, in the various perihepatic spaces, within the lesser sac, or along the major routes of communication between intraperitoneal recesses, such as the right paracolic gutter. Moreover, infection may be contained within the intra-abdominal viscera, as in hepatic, pancreatic, splenic, tuboovarian, or renal abscess. Abscesses also frequently form around diseased viscera and between adjacent loops of the bowel [1,2,3].

IAI is frequently endogenous in origin and is generally caused by the large number and variety of microorganisms that colonizes mucous membranes lining certain viscera within the abdominal cavity. Notably, the mucous membranes of the stomach, upper small bowel, lower small bowel, and large bowel, as well as the genitourinary tract, each have characteristic microflora in terms of type of microbial species, total number of different species, and microbial density. Consequently, the most common bacterial organisms involved with IAI are Enterobacterales and anaerobes from the endogenous microbiota. Initial antimicrobial therapy is frequently empirical and inappropriate empirical therapy is correlated with increased risk of clinical failure, increased time of hospitalization, and markedly higher costs [4,5,6].

These infections usually require both surgical intervention and rapid initiation of effective antibiotic therapy Standard antimicrobial regimens for the treatment of IAIs often include a β-lactam such as piperacillin-tazobactam, broad-spectrum cephalosporin or carbapenem. Nonetheless, the emergence and dissemination of Enterobacterales that produce extended-spectrum β-lactamases (ESBLs) and/or carbapenemases represent important therapeutic challenges by limiting the options of effective antimicrobials [1,2,3].

Various β-lactamase inhibitor combinations (BLICs) targeting multidrug-resistant (MDR) Enterobacterales were approved for clinical use in recent years, such as ceftazidime-avibactam, ceftolozane-tazobactam, imipenem-relebactam, and meropenem-vaborbactam [7]. These agents represent a notable advancement in the management of infections caused by MDR Gram-negatives, especially ESBL producers and carbapenem-resistant Enterobacterales (CRE). Nonetheless, increasing resistance to these agents has been reported in some US regions [8].

Aztreonam-avibactam was recently approved by the European Medicines Agency (EMA) in the European Union for the treatment of adults with complicated IAI, hospital-acquired pneumonia, ventilator-associated pneumonia, and complicated urinary tract infection (cUTI) (https://www.ema.europa.eu/en/news/new-antibiotic-fight-infections-caused-multidrug-resistant-bacteria; accessed on 20 February 2025) and by the US Food and Drug Administration (FDA) for treatment of complicated IAI [9]. In this investigation, we evaluated the antimicrobial susceptibility and β-lactamase profile of Enterobacterales collected from patients with IAI hospitalized in US medical centres with focus on the activity of newer BLICs active against MDR Enterobacterales and CRE, including aztreonam-avibactam, ceftazidime-avibactam, meropenem-vaborbactam, and imipenem-relebactam.

## 2. Results

*E. coli* was the most common Enterobacterales species (47.1% of isolates), followed by *K. pneumoniae* (18.7%), and *E. cloacae* species complex (9.8%). Aztreonam-avibactam (MIC_50/90_, ≤0.03/0.12 mg/L), ceftazidime-avibactam (MIC_50/90_, 0.12/0.25 mg/L), and meropenem-vaborbactam (MIC_50/90_, 0.03/0.06 mg/L) were the most active agents with 99.9% susceptibility (Table 1 and Table 2). Meropenem (MIC_50/90_, 0.03/0.06 mg/L) was also highly active with 99.2% of isolates inhibited at the CLSI susceptible breakpoint. Among BLICs, imipenem-relebactam (MIC_50/90_, 0.12/0.5 mg/L; 96.1%/99.5% susceptible per CLSI/EUCAST) and ceftolozane-tazobactam (MIC_50/90_, 0.25/1 mg/L; 93.0% susceptible) were active against >90% of isolates, piperacillin-tazobactam (MIC_50/90_, 2/16 mg/L) retained activity against 87.2% of isolates, and ampicillin-sulbactam (MIC_50/90_, 8/64 mg/L; 51.6% susceptible per CLSI criteria) exhibited limited activity against Enterobacterales isolated from patients with IAI. (Table 1 and Table 2).

Cefepime (MIC_50/90_, 0.06/0.25 mg/L; 89.4%/87.4% susceptible per CLSI/EUCAST) was slightly more active than ceftazidime (MIC_50/90_, 0.25/32 mg/L; 85.6%/81.4%% susceptible per CLSI/EUCAST) and ceftriaxone (MIC_50/90_, ≤0.06/>8 mg/L; 81.6% susceptible per CLSI and EUCAST) and the aminoglycosides gentamicin (MIC_50/90_, 0.5/1 mg/L; 92.7% susceptible per CLSI and EUCAST) and amikacin (MIC_50/90_, 2/4 mg/L; 95.3%/98.9% susceptible per CLSI/EUCAST) were very active against the Enterobacterales isolated from patients with IAI (Table 1).

**Figure 1:**
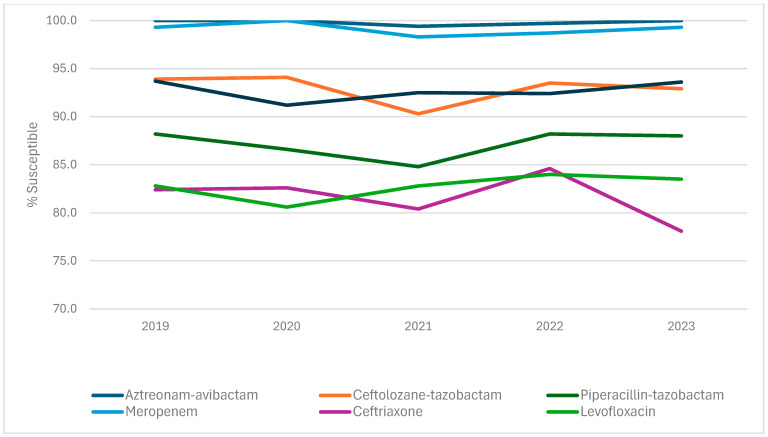
Yearly trend of Enterobacterales (*n* = 2036) susceptibility to selected antimicrobial agents.

Susceptibility rates remained stable for most antimicrobial agents during the 5 years of the investigation (Table 3 and Figure 1). Notably, susceptibility to aztreonam-avibactam, ceftazidime-avibactam, and meropenem-vaborbactam remained >99% every year and susceptibility to meropenem ranged from 98.7% (2022) to 100.0% (2020; Table 3).

An acquired broad-spectrum β-lactamase gene was identified in 207 (10.2%) isolates and included ESBL genes (*n* = 182), transferable AmpC genes (*n* = 24), and carbapenemase genes (*n* = 9; Table 4). Eight isolates produced 2 β-lactamase classes, including an ESBL plus a transferable AmpC (*n* = 4) and an ESBL plus a carbapenemase (*n* = 4). The most common ESBL genes were *bla*_CTX-M_ type (170 isolates), *bla*_OXA-1/30_ (63 isolates; all also carried a *bla*_CTX-M_), and *bla*_SHV_ type (14 isolates). Sixty-five isolates had 2 ESBL genes. A transferable AmpC gene was identified on 24 isolates, including 22 *bla*_CMY_ type and 2 *bla*_DHA-1_; a carbapenemase gene was found on 9 isolates (60.0% of CREs), including *bla*_KPC-2_ (3 isolates), *bla*_KPC-3_ (4 isolates), and *bla*_NDM-5_ (2 isolates; Table 4).

Aztreonam-avibactam (MIC_50/90_, 0.06/0.25 mg/L; 99.0% susceptible), ceftazidime-avibactam (MIC_50/90_, 0.12/0.5 mg/L; 99.0% susceptible), meropenem-vaborbactam (MIC_50/90_, 0.03/0.03 mg/L; 99.0% susceptible), and imipenem-relebactam (MIC_50/90_, 0.12/0.12 mg/L; 98.4% susceptible) were the most active compounds against the β-lactamase producer group; whereas ceftolozane-tazobactam (MIC_50/90_, 0.5/16 mg/L; 82.0% susceptible) and piperacillin-tazobactam (MIC_50/90_, 4/>128 mg/L; 70.0% susceptible) showed limited activity against these organisms (Table 1 and Table 2). Other compounds active against >90% of β-lactamase producers were meropenem (MIC_50/90_, 0.03/0.06 mg/L; 94.2% susceptible) and tigecycline (MIC_50/90_, 0.25/1 mg/L; 96.6%/89.9% susceptible per US FDA/EUCAST criteria. Amikacin (MIC_50/90_, 2/8 mg/L) was active against 83.1%/92.3% of β-lactamase producers per CLSI/EUCAST criteria, whereas gentamicin (MIC_50/90_, 1/>16 mg/L; 63.8% susceptible per CLSI and EUCAST) and levofloxacin (MIC_50/90_, 8/32 mg/L; 32.9% susceptible per CLSI and EUCAST) exhibited limited activity against these organisms (Table 1).

Aztreonam-avibactam, ceftazidime-avibactam, meropenem-vaborbactam, and imipenem-relebactam also retained good activity against piperacillin-tazobactam–nonsusceptible (98.7–99.2% susceptible) and CRE isolates (86.7% (13/15) susceptible for all 4 compounds; Table 2). Cefiderocol, which was only tested against CRE isolates, was active against 93.3% (14/15) and 80.0% (12/15) of CRE isolates per CLSI and EUCAST criteria, respectively (Table 1). CRE susceptibility to amikacin (MIC_50/90_, 4/32 mg/L) and tigecycline (MIC_50/90_, 0.5/1 mg/L) varied markedly according to the breakpoint criteria applied. Amikacin was active against 73.3%/86.7% of isolates per CLSI/EUCAST criteria and tigecycline was active against 100.0%/80.0% of isolates per US FDA/EUCAST criteria (Table 1).

Only 3 isolates (0.1%) exhibited aztreonam-avibactam MICs >4 mg/L: 2 carbapenem-resistant *E. coli* isolates with MIC values of 8 and 16 mg/L and a carbapenem-susceptible/intermediate *E. cloacae* with an aztreonam-avibactam MIC of 8 mg/L. The WGS results showed insertions in the PBP3 gene of both *E. coli* isolates (E149D, T233A, V332I, P333_Y334insYRIK, A413V) and the *E. cloacae* isolate had alterations in OmpC (L211I, G228R) and OmpF (Y48N, A49D, G50S, D181T, D200E) genes with no major alterations in the PBP3 gene.

## 3. Discussion

The selection of initial antibiotic therapy for IAI is generally empiric, and failure to start an effective antimicrobial regimen early during treatment leads to an increased risk of clinical failure [6]. Although broad-spectrum regimens could be more effective, their advantages must be assessed against the potential risk of the development of antimicrobial resistance. Prompt initiation of effective antimicrobial treatment in patients with life-threatening infections, such as complicated IAI and sepsis, has a substantial influence on the clinical outcome, the need for surgical intervention, length of hospitalization, selection of resistant organisms, and overall healthcare costs [1,4].

The guidelines for the treatment of IAI published by the Infectious Disease Society of America (IDSA) [1], the Surgical Infection Society [2], and the European Society of Clinical Microbiology and Infections Diseases [3] are comprehensive documents that provide evidence-based recommendations for the diagnosis and management of patients with complicated and uncomplicated IAI. Moreover, because initial antibiotic therapy is frequently empiric, results of large surveillance programmes provide valuable information to guide the proper choice of empiric therapy, especially where local data are not available. Unfortunately, surveillance results on the antimicrobial susceptibly of bacteria causing IAI in US medical centres are scarce [12].

The results of antimicrobial susceptibility and β-lactamase profile of a large collection of Enterobacterales (*n* = 2036) isolated from patients with IAI in 63 US medical centres over a period of 5 years (2019–2023) presented here revealed some interesting results. The BLICs aztreonam-avibactam, ceftazidime-avibactam, and meropenem-vaborbactam exhibited almost complete activity (99.9% susceptibility). One *E. coli* strain was resistant to these three compounds, one *E. coli* strain was resistant to ceftazidime-avibactam and meropenem-vaborbactam, while another *E. coli* and two *E. cloacae* isolates were resistant to only one of these three compounds. Susceptibility to imipenem-relebactam (96.1%) was lower than aztreonam-avibactam, ceftazidime-avibactam, meropenem-vaborbactam, and even meropenem (99.2% susceptible) due to the limited activity of this compound against organisms of Morganellaceae family [13]. Most imipenem-relebactam-nonsusceptible isolates (50 of 52) were *Proteus mirabilis*, *P. vulgaris* group, and *Morganella morganii*.

We observed a high prevalence of isolates producing β-lactamases that hydrolyses broad-spectrum β-lactams, such as ESBLs, transferable AmpC, and carbapenemases (10.2%). Our results corroborate other investigations by showing a lower frequency of Enterobacterales producing carbapenemases and AmpC when compared to ESBLs and the continued predominance of CTX-M among ESBL producers in US medical centres [14,15]. Among the antimicrobial agents tested, only aztreonam-avibactam, ceftazidime-avibactam, meropenem-vaborbactam, imipenem-relebactam, meropenem, and tigecycline exhibited good activity against these two resistant subsets. Although the prevalence of CRE was relatively low (0.7%), the emergence of resistance to the new BLICs and cefiderocol among these organisms is of great concern.

The analysis and interpretation of the results presented here should consider the limitations of the study. The main limitation of the study was the fact that the investigation was limited to Enterobacterales; thus, we were not able to evaluate the etiology of IAI in US medical centres. The criteria to categorize an organism as the probable cause of the infection is another limitation of the study since it may vary among the participant centres and a small number of organisms that were colonizers or contaminants could have been included in the study. The lack of differentiation between complicated and uncomplicated IAI is certainly another limitation of the study.

## 4. Conclusions

The results of the present study highlight the value of comprehensive surveillance programmes on the monitorization of resistance phenotypes and resistance mechanisms. Due to the rapid changes in the epidemiology of β-lactam resistance mechanisms, the results of surveillance programmes are critical in designing infection control measures and determining empiric antibiotic therapy guidelines.

## 5. Materials and Methods

### 5.1. Organism Collection

A total of 2036 Enterobacterales isolates were consecutively collected from patients with IAI in 63 US medical centres (33 states) in 2019–2023 via a network of medical sites participating in the International Network for Optimal Resistance Monitoring (INFORM) Surveillance Program and sent to Element Iowa City (JMI Laboratories; North Liberty, IA, USA) for susceptibility testing [15]. Each participating centre was invited to collect a specific number of consecutive bacterial isolates (any species) each year from patients hospitalized with IAI. Only bacterial isolates determined to be significant by local criteria as the reported probable cause of infection were included in the study.

### 5.2. Susceptibility Testing

All isolates were susceptibility tested by the broth microdilution method specified by Clinical Laboratory Standard Institute (CLSI) standards [16] at Element Iowa City (JMI Laboratories). Upon receipt from the participant centre isolates were subcultured and checked for purity. Validated frozen form MIC panels were manufactured at Element Iowa City (JMI Laboratories). The following antimicrobial agents were tested (dilution ranges are shown in parenthesis): aztreonam-avibactam (0.03 to 16 mg/L at fixed avibactam concentration of 4 mg/L), ceftazidime-avibactam (0.015 to 32 mg/L at fixed avibactam concentration of 4 mg/L), meropenem-vaborbactam (0.015 to 32 mg/L at fixed vaborbactam concentration of 8 mg/L), imipenem-relebactam (0.03 to 16 mg/L at fixed relebactam concentration of 4 mg/L), ceftolozane-tazobactam (0.12 to 16 mg/L at fixed tazobactam concentration of 4 mg/L), piperacillin-tazobactam (0.25 to 128 mg/L at fixed tazobactam concentration of 4 mg/L), ampicillin-sulbactam (0.5 to 64 mg/L at2:1 ratio), aztreonam (0.03 to 16 mg/L), ceftriaxone (0.03 to 8 mg/L), ceftazidime (0.015 to 32 mg/L), cefepime (0.03 to 32 mg/L), meropenem (0.015 to 32 mg/L), imipenem (0.03 to 8 mg/L), levofloxacin (0.015 to 32 mg/L), gentamicin (0.12 to 16 mg/L), amikacin (0.25 to 32 mg/L), tigecycline (0.06 to 8 mg/L), colistin (0.06 to 8 mg/L) [16]. Cefiderocol (0.004 to 64 mg/L) was tested on iron-depleted media against CRE isolates only [16]. Microdilution panels were inoculated within 2 h of complete thawing.

The aztreonam-avibactam susceptible breakpoint published by EUCAST and proposed by the FDA (≤4 mg/L) was applied [11]. CRE was defined as demonstrating imipenem or meropenem MIC values of ≥4 mg/L. CLSI [10] and US FDA breakpoints (https://www.fda.gov/drugs/development-resources/antibacterial-susceptibility-test-interpretive-criteria) (accessed on 1 April 2025) were applied to the comparator agents where available. Concurrent quality control (QC) testing of *E. coli* ATCC 25922, *K. pneumoniae* ATCC 700,603 and BAA-2814, and *P. aeruginosa* ATCC 27,853 and JMI SR27001 was performed to ensure proper test conditions and procedures.

### 5.3. Βeta-Lactamase Screening

The following organism subsets were selected for whole genome sequencing (WGS): (1) *Escherichia coli*, *Klebsiella pneumoniae*, *K. oxytoca*, and *Proteus mirabilis* isolates displaying MIC values ≥ 2 mg/L for at least 2 of the following β-lactams: ceftazidime, ceftriaxone, aztreonam, or cefepime; (2) *Enterobacter cloacae* species complex (herein *E. cloacae*) and *Citrobacter* spp. isolates displaying MIC values ≥ 16 mg/L for ceftazidime and/or ≥2 mg/L for cefepime; (3) Enterobacterales isolates displaying elevated carbapenem (imipenem and/or meropenem) MIC results > 1 mg/L (*Proteus mirabilis* or indole-positive Proteeae included if meropenem MIC was >1 mg/L); (4) Enterobacterales isolates displaying aztreonam-avibactam MIC values > 4 mg/L.

Total genomic DNA was extracted using the fully automated Thermo Scientific™ KingFisher™ Flex Magnetic Particle Processor (Cleveland, OH, USA). DNA extracts were quantified using the Qubit™ High Sensitivity DS-DNA assay (Invitrogen, ThermoFisher Inc., Waltham, MA, USA) and normalized to 0.2 ng/µL. A total of 1 ng high-quality genomic DNA was used as input material for library construction using the Nextera XT™ DNA library preparation kit (Illumina, San Diego, CA, USA). Libraries were normalized using the bead-based normalization procedure (Illumina) and sequenced on MiSeq. The generated FASTQ files were assembled using SPAdes Assembler and subjected to proprietary software (Element Iowa City [JMI Laboratories]) for screening of β-lactamase genes [17]. An in-house proprietary bioinformatic pipeline and a JMI Laboratories-curated resistance gene database (Version 3; uses Python v2.7.9, SPAdes v3.15.3, and BBMap v36.x) based on the NCBI Bacterial Antimicrobial Resistance Reference Gene Database (https://www.ncbi.nlm.nih.gov/bioproject/PRJNA313047) (accessed on 1 December 2024) was used for the in silico analysis of acquired resistance genes. Genes encoding intrinsic resistance factors (e.g., amino acid alterations in OmpC, OmpF, and PBP3) were assessed relative to a susceptible reference strain [15,18].

### 5.4. Characterization of Aztreonam-Avibactam Resistant Isolates

Isolates with aztreonam-avibactam MIC values > 4 mg/L (nonsusceptible per US FDA and EUCAST) were molecularly characterized by extraction of sequences encoding acquired and intrinsic resistance genes (e.g., ESBL, carbapenemase, and chromosomal *ampC* genes). Furthermore, the amino acids sequences of penicillin-binding protein 3 (FtsI) and the porins OmpC/OmpF (*E. coli*, and *S. marcescens*) and the homologoous OmpK36/OmpK35 (*K. aerogenes*, *K. oxytoca*) were compared to the corresponding sequences from the susceptible reference strains *E. coli* ATCC 25922, *K. aerogenes* KCTC2190 (PBP3)/ATCC 13,048 (OmpK36)/OmpK35 (ATCC 15038), *K. oxytoca* KCTC1686 [19].

## Figures and Tables

**Table 1:** Antimicrobial susceptibility of Enterobacterales and selected resistant subsets.

Antimicrobial Agent (No. of Isolates)	mg/L		CLSI ^a^			EUCAST ^a^		
	MIC_50_	MIC_90_	%S	%I	%R	%S	%I	%R
All Enterobacterales (2036)								
Aztreonam-avibactam	≤0.03	0.12	99.9 ^b^	0.1	<0.1	99.9		0.1
Ceftazidime-avibactam	0.12	0.25	99.9		0.1	99.9		0.1
Meropenem-vaborbactam	0.03	0.06	99.9	0.0	0.1	99.9		0.1
Imipenem-relebactam ^c^	0.12	0.5	96.1	3.4	0.5	99.5		0.5
Ceftolozane-tazobactam	0.25	1	93.0	1.3	5.7	93.0		7.0
Piperacillin-tazobactam	2	16	87.2	2.9 ^d^	9.9	87.2		12.8
Ampicillin-sulbactam	8	64	51.6	16.3	32.1			
Aztreonam	0.12	>16	84.6	2.1	13.3	82.3	2.3	15.4
Ceftriaxone	≤0.06	>8	81.6	0.8	17.6	81.6	0.8	17.6
Ceftazidime	0.25	32	85.6	1.4	13.1	81.4	4.2	14.4
Cefepime	0.06	4	89.4	3.3 ^d^	7.3	87.4	3.7	8.8
Meropenem	0.03	0.06	99.2	0.1	0.7	99.3	0.3	0.3
Imipenem	≤0.12	1	93.9	4.6	1.5	98.5	0.9	0.6
Levofloxacin	0.06	8	82.7	1.9	15.3	82.7	1.9	15.3
Gentamicin	0.5	1	92.7	0.3	6.9	92.7 ^e^		7.3
Amikacin	2	4	95.3	3.6	1.1	98.9 ^e^		1.1
Tigecycline	0.25	1	97.2 ^b^	2.7	<0.1	86.3 ^f^		13.7
Colistin	0.25	>8		87.0	13.0	87.0		13.0
MDR (436) ^g^								
Aztreonam-avibactam	0.06	0.5	99.3 ^b^	0.5	0.2	99.3		0.7
Ceftazidime-avibactam	0.25	1	99.3		0.7	99.3		0.7
Meropenem-vaborbactam	0.03	0.06	99.5	0.0	0.5	99.5		0.5
Imipenem-relebactam ^c^	0.12	0.25	99.4	0.0	0.6	99.4		0.6
Ceftolozane-tazobactam	0.5	16	67.9	5.7	26.4	67.9		32.1
Piperacillin-tazobactam	8	>128	50.2	8.3 ^d^	41.5	50.2		49.8
Ampicillin-sulbactam	64	>64	4.6	17.9	77.5			
Aztreonam	>16	>16	35.3	6.4	58.3	30.5	4.8	64.7
Ceftriaxone	>8	>8	29.4	1.6	69.0	29.4	1.6	69.0
Ceftazidime	16	>32	38.8	4.6	56.7	31.2	7.6	61.2
Cefepime	2	>32	56.0	12.6 ^d^	31.4	47.9	14.9	37.2
Meropenem	0.03	0.12	96.1	0.7	3.2	96.8	1.6	1.6
Levofloxacin	1	32	46.9	4.6	48.5	46.9	4.6	48.5
Gentamicin	0.5	>16	70.2	0.9	28.9	70.2 ^e^		29.8
Amikacin	2	8	88.1	7.6	4.4	95.6 ^e^		4.4
Tigecycline	0.25	1	95.9 ^b^	4.1	0.0	86.5 ^f^		13.5
Colistin	0.25	0.5		93.8	6.2	93.8		6.2
β-lactamase producers (207) ^h^								
Aztreonam-avibactam	0.06	0.25	99.0 ^b^	0.5	0.5	99.0		1.0
Ceftazidime-avibactam	0.12	0.5	99.0		1.0	99.0		1.0
Meropenem-vaborbactam	0.03	0.03	99.0	0.0	1.0	99.0		1.0
Imipenem-relebactam ^c^	0.12	0.12	98.4	0.0	1.6	98.4		1.6
Ceftolozane-tazobactam	0.5	16	82.0	2.4	15.5	82.0		18.0
Piperacillin-tazobactam	4	>128	70.0	8.2 ^d^	21.7	70.0		30.0
Ampicillin-sulbactam	32	>64	16.4	17.4	66.2			
Aztreonam	>16	>16	9.7	14.0	76.3	0.5	9.2	90.3
Ceftriaxone	>8	>8	0.0	0.0	100.0	0.0	0.0	100.0
Ceftazidime	16	>32	20.3	9.7	70.0	2.9	17.4	79.7
Cefepime	32	>32	17.4	16.9 ^d^	65.7	13.0	12.1	74.9
Meropenem	0.03	0.06	94.2	0.5	5.3	94.7	1.9	3.4
Levofloxacin	8	32	32.9	4.8	62.3	32.9	4.8	62.3
Gentamicin	1	>16	63.8	1.4	34.8	63.8 ^e^		36.2
Amikacin	2	8	83.1	9.2	7.7	92.3 ^e^		7.7
Tigecycline	0.25	1	96.6 ^b^	3.4	0.0	89.9 ^f^		10.1
Colistin	0.25	0.25		99.0	1.0	99.0		1.0
CRE (15)								
Aztreonam-avibactam	0.5	8	86.7 ^b^	6.7	6.7	86.7		13.3
Ceftazidime-avibactam	1	32	86.7		13.3	86.7		13.3
Meropenem-vaborbactam	0.5	32	86.7	0.0	13.3	86.7		13.3
Imipenem-relebactam	0.12	4	86.7 ^d^	0.0	13.3	86.7 ^d^		13.3
Cefiderocol ^i^	2	4	93.3	0.0	6.7	80.0		20.0
Levofloxacin	16	32	40.0	6.7	53.3	40.0	6.7	53.3
Gentamicin	1	16	80.0	6.7	13.3	80.0		20.0
Amikacin	4	32	73.3	13.3	13.3	86.7		13.3
Tigecycline	0.5	1	100.0	0.0	0.0	80.0		20.0

^a^ Criteria as published by CLSI (2025) [10] and EUCAST (2025) [11]. ^b^ Breakpoints published by the US Food and Drug Administration (FDA) were applied. ^c^ All Enterobacterales species were included in the analysis, but CLSI and EUCAST exclude organisms from the family Morganellaceae. ^d^ Intermediate is interpreted as susceptible-dose dependent. ^e^ For infections originating from the urinary tract. For systemic infections, aminoglycosides must be used in combination with other active therapy. ^f^ EUCAST breakpoints for *Escherichia coli* and *Citrobacter koseri* were applied. ^g^ Isolates nonsusceptible to at least 1 drug in ≥3 classes. Organisms include *Citrobacter amalonaticus* (1), *C. amalonaticus/farmeri* (6), *C. freundii* species complex (79), *C. koseri* (22), *Enterobacter cloacae* species complex (199), *Escherichia coli* (958), *E. hermannii* (1), *Hafnia alvei* (8), *Klebsiella aerogenes* (41), *K. oxytoca* (88), *K. pneumoniae* (380), *K. variicola* (21), *Kluyvera ascorbata* (1), *Leclercia adecarboxylata* (1), *Lelliottia amnigena* (1), *Morganella morganii* (33), *Pantoea agglomerans* (1), *P. eucrina* (1), *Pluralibacter gergoviae* (1), *Proteus hauseri* (1), *P. mirabilis* (80), *P. penneri* (4), *P. vulgaris* (9), *P. vulgaris* group (8), *Providencia alcalifaciens* (1), *P. rettgeri* (2), *P. stuartii* (1), *Raoultella ornithinolytica* (7), *R. planticola* (1), *Serratia marcescens* (65), *S. rubidaea* (1), unspeciated *Citrobacter* (1), *unspeciated Cronobacter* (2), unspeciated *Pantoea* (1), and unspeciated *Raoultella* (9). ^h^ Include isolates producing an ESBL (*n* = 182), a transferable AmpC (24), or a carbapenemase (*n* = 9). Eight isolates produced 2 β-lactamase classes, including an ESBL plus a transferable AmpC (*n* = 3) and an ESBL plus a carbapenemase (*n* = 5). Organisms include *Citrobacter freundii* species complex (1), *Enterobacter cloacae* species complex (10), *Escherichia coli* (148), *Klebsiella oxytoca* (1), and *K. pneumoniae* (47). ^i^ Cefiderocol was only tested against carbapenem-resistant Enterobacterales (CRE). Abbreviations: MDR, multidrug-resistant; CRE, carbapenem-resistant Enterobacterales.

**Table 2:** Activity of β-lactamase inhibitor combinations and ceftriaxone against Enterobacterales from intra-abdominal infections.

Organism/Group		% Susceptible ^a^ (MIC_50/90_ in mg/L)						
(No. of Isolates)	ATM-AVI	CAZ-AVI	MEM-VAB	IMI-REL	TOL-TAZ	PIP-TAZ	AMP-SUL	Ceftriaxone
Enterobacterales (2036)	99.9 (≤0.03/0.12)	99.9 (0.12/0.25)	99.9 (0.03/0.06)	96.1 (0.12/0.5)	93.0 (0.25/1)	87.2 (2/16)	51.6 (8/64)	81.6 (≤0.06/>8)
MDR (436)	99.3 (0.06/0.5)	99.3 (0.25/1)	99.5 (/0.030.06)	99.2 (0.12/0.25)	67.9 (0.5/16)	50.2 (8/>128)	4.6 (64/>64)	29.4 (>8/>8)
PIP-TAZ-NS (260)	98.8 (0.12/1)	98.8 (0.5/1)	99.2 (0.03/0.06)	98.7 (0.12/0.25)	46.2 (4/>16)	--	1.9 (>64/>64)	28.1 (>8/>8)
β-Lactamase producers (207) ^b^	99.0 (0.06/0.25)	99.0 (0.12/0.5)	99.0 (0.03/0.03)	98.4 (0.12/0.12)	82.0 (0.5/16)	70.0 (4/>128)	16.4 (32/>64)	0.0 (>8/>8)
CRE (15)	86.7 (0.5/8)	86.7 (1/32)	86.7 (0.5/32)	86.7 (0.12/4)	0.0 (>16/>16)	0.0 (>128/>128)	0.0 (>64/>64)	0.0 (>8/>8)
*E. coli* (958)	99.8 (≤0.03/0.12)	99.8 (0.12/0.25)	99.8 (≤0.015/0.03)	99.6 (0.12/0.12)	97.9 (0.25/0.5)	93.4 (2/8)	54.2 (8/64)	83.6 (≤0.06/>8)
*K. pneumoniae* (380)	100.0 (≤0.03/0.12)	100.0 (0.12/0.25)	100.0 (0.03/0.03)	100.0 (0.12/0.25)	96.6 (0.25/1)	86.8 (4/16)	73.2 (8/64)	87.4 (≤0.06/>8)
*E. cloacae* complex (199)	99.5 (0.06/1)	99.5 (0.25/1)	100.0 (0.03/0.06)	100.0 (0.12/0.25)	67.0 (0.5/16)	61.3 (4/>128)	12.6 (64/>64)	57.3 (0.5/>8)
*Morganellaceae* (135) ^c^	100.0 (≤0.03/≤0.03)	100.0 (0.06/0.06)	100.0 (0.06/0.12)	35.8 (2/2)	99.3 (0.5/0.5)	100.0 (0.25/1)	59.3 (2/32)	85.2 (≤0.06/8)
*K. oxytoca* (88)	100.0 (≤0.03/0.06)	100.0 (0.12/0.25)	100.0 (0.03/0.03)	100.0 (0.12/0.25)	97.7 (0.25/0.5)	94.3 (2/8)	34.1 (16/32)	90.9 (≤0.06/0.5)
*C. freundii* complex (79)	100.0 (0.06/0.25)	100.0 (0.12/0.5)	100.0 (0.03/0.03)	100.0 (0.12/0.12)	75.6 (0.25/16)	69.6 (4/128)	57.0 (8/>64)	72.2 (0.25/>8)
*S. marcescens* (65)	100.0 (0.12/0.25)	100.0 (0.25/0.5)	100.0 (0.06/0.06)	100.0 (0.5/1)	96.9 (0.5/1)	92.3 (2/8)	7.7 (64/>64)	89.2 (0.5/2)
Other species (132) ^d^	100.0 (0.06/0.25)	100.0 (0.12/0.5)	100.0 (0.03/0.06)	100.0 (0.12/0.25)	84.8 (0.25/8)	72.7 (4/64)	51.5 (8/64)	78.8 (0.12/>8)

^a^ % susceptible per CLSI criteria except for aztreonam-avibactam where EUCAST criteria (susceptible at ≤4 mg/L) was applied [11]. ^b^ Include isolates producing an ESBL (*n* = 182), transferable AmpC (24), carbapenemase (*n* = 9). Eight isolates produced 2 β-lactamase classes, including an ESBL plus a transferable AmpC (*n* = 3) and an ESBL plus a carbapenemase (*n* = 5). ^c^ Includes: *Morganella morganii* (33), *Proteus hauseri* (1), *P. mirabilis* (80), *P. vulgaris* (9), *P. vulgaris* group (8), *Providencia alcalifaciens* (1), *P. rettgeri* (2), and *P. stuartii* (1). ^d^ Includes: Citrobacter amalonaticus (1), C. amalonaticus/farmeri (5), C. farmeri (1), C. koseri (22), Escherichia hermannii (1), Hafnia alvei (8), Klebsiella aerogenes (41), K. variicola (21), Kluyvera ascorbata (1), Leclercia adecarboxylata (1), Lelliottia amnigena (1), Pantoea agglomerans (1), P. eucrina (1), Pluralibacter gergoviae (1), Proteus penneri (4), Raoultella ornithinolytica (7), R. planticola (1), Serratia rubidaea (1), unspeciated Citrobacter (1), unspeciated Cronobacter (2), unspeciated Pantoea (1), and unspeciated Raoultella (9). Abbreviations: ATM-AVI, aztreonam-avibactam; CAZ-AVI, ceftazidime-avibactam; MEM-VAB, meropenem-vaborbactam; IMI-REL, imipenem-relebactam; TOL-TAZ, ceftolozane/tazobactam; PIP-TAZ, piperacillin-tazobactam; AMP-SUL, ampicillin-sulbactam; CRE, carbapenem-resistant Enterobacterales; MDR, multidrug-resistant.

**Table 3:** Antimicrobial susceptibility of Enterobacterales (*n* = 2036) to selected antimicrobial agents stratified by year.

Antimicrobial	% Susceptible (CLSI) by Year				
agent	2019	2020	2021	2022	2023
Aztreonam-avibactam	100.0	100.0	99.4	99.7	100.0
Ceftazidime-avibactam	100.0	100.0	99.4	99.7	100.0
Meropenem-vaborbactam	100.0	100.0	99.7	99.7	100.0
Imipenem-relebactam	- ^a^	- ^a^	94.1	96.3	95.5
Ceftolozane-tazobactam	93.9	94.1	90.3	93.5	92.9
Piperacillin-tazobactam	88.2	86.6	84.8	88.2	88.0
Meropenem	99.3	100.0	98.3	98.7	99.3
Ceftriaxone	82.4	82.6	80.4	84.6	78.1
Levofloxacin	82.8	80.6	82.8	84.0	83.5
Gentamicin	93.7	91.2	92.5	92.4	93.6

^a^ Imipenem-relebactam was tested only in 2021–2023.

**Table 4:** Extended-spectrum β-lactamase, transferable AmpC, and carbapenemases identified among Enterobacterales from patients with intra-abdominal infections.

Organisms/β-Lactamase	No. of Isolates
*Citrobacter freundii* species complex	1
CMY-181	1
*Enterobacter cloacae* species complex	10
CTX-M-15	1
CTX-M-15, OXA-1/30	3
SHV-12	5
KPC-2	1
*Escherichia coli*	148
CMY-2	16
CMY-2, CTX-M-15	1
CMY-42, CTX-M-15, OXA-1/30	1
CMY-44-like, CTX-M-27	1
CTX-M-1	1
CTX-M-115	1
CTX-M-14	11
CTX-M-15	25
CTX-M-15, CTX-M-33	1
CTX-M-15, NDM-5, OXA-1/30	2
CTX-M-15, OXA-1/30	38
CTX-M-15, OXA-1/30, TEM-169	1
CTX-M-24	2
CTX-M-27	30
CTX-M-55	13
CTX-M-64	1
DHA-1	1
SHV-7	1
KPC-3	1
*Klebsiella oxytoca*	1
KPC-2	1
*Klebsiella pneumoniae*	47
CMY-2	2
CTX-M-15	14
CTX-M-15, OXA-1/30	17
CTX-M-15, OXA-1/30, SHV-7	1
CTX-M-15, SHV-27	1
CTX-M-27	1
CTX-M-3	3
KPC-2, SHV-12	1
KPC-3	2
KPC-3, SHV-12	1
SHV-12, DHA-1	1
SHV-27	1
SHV-2-like	1
SHV-7	1
Grand Total	207

## Data Availability

Data for the SENTRY Program is available at: www.sentry-mvp.jmilabs.com. Molecular data is unavailable due to privacy since some of the isolates are being further characterized for ongoing studies.
